# Selection of a Full Agonist Combinatorial Antibody that Rescues Leptin Deficiency In Vivo

**DOI:** 10.1002/advs.202000818

**Published:** 2020-07-01

**Authors:** Pingdong Tao, Yuanyuan Kuang, Yu Li, Wenping Li, Zibei Gao, Lili Liu, Min Qiang, Zhao Zha, Kun Fan, Peixiang Ma, Jeffrey M. Friedman, Guang Yang, Richard A. Lerner

**Affiliations:** ^1^ Shanghai Institute for Advanced Immunochemical Studies ShanghaiTech University Shanghai 201210 China; ^2^ School of Life Science and Technology ShanghaiTech University Shanghai 201210 China; ^3^ Institute of Biochemistry and Cell Biology Shanghai Institutes for Biological Sciences Chinese Academy of Sciences Shanghai 200031 China; ^4^ University of Chinese Academy of Sciences Beijing 100049 China; ^5^ Laboratory of Molecular Genetics Howard Hughes Medical Institute The Rockefeller University New York NY 10065 USA; ^6^ Department of Chemistry Scripps Research Institute La Jolla CA 92037 USA

**Keywords:** combinatorial antibody library, full agonists, function‐guided selection, leptin receptors, replacement therapy

## Abstract

Growth factor deficiency in adulthood constitutes a distinct clinical syndrome with significant morbidities including abnormal body composition, reduced energy, affective disturbances, dyslipidemia, and increased cardiovascular risk. Protein replacement therapies using recombinant proteins or enzymes represent the only approved treatment. Combinatorial antibodies have shown great promise as a new class of therapeutic molecules because they act as “mechanism‐based antibodies” with both agonist and antagonist activities. Using leptin, a key hormone in energy metabolism, as an example, a function‐guided approach is developed to select combinatorial antibodies with high potency and full agonist activity that substitute natural growth factors in vivo. The identified antibody shows identical biochemical properties and cellular profiles as leptin, and rescues leptin‐deficiency in ob/ob mice. Remarkably, the antibody activates leptin receptors that are otherwise nonfunctional because of mutations (L372A and A409E). Combinatorial antibodies have significant advantages over recombinant proteins for chronical usage in terms of immunological tolerance and biological stability.

## Graphical abstract

A full agonist antibody against leptin receptor is discovered using a function‐guided approach from a combinatorial antibody library. It shows identical biochemical properties and functional profiles as leptin both in vitro and in vivo. Furthermore, it activates several leptin receptor mutants which cannot be activated by leptin, indicating promising therapeutic applications. The selection strategy can be applied to other cellular targets.

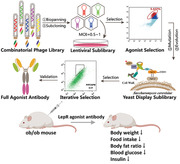

## 1 Introduction

Growth factors and cytokines are critical modulators of physiological processes important to the body's homeostasis.^[^ ^1^, ^2^, ^3^ ^]^ They are external signaling molecules exerting physiological functions through interaction with their cognate receptors on cell surfaces. Based on their functions and structures, growth factors are categorized into different protein families such as, interleukins, interferons, tumor necrosis factors (TNFs), fibroblast growth factors (FGFs), epidermal growth factors (EGFs), vascular endothelial growth factors (VEGFs), hepatocyte growth factors (HGFs), transforming growth factors (TGFs), hematopoietic factors, and neurotrophic factors, to name a few.^[^ ^4^ ^]^ The first approved recombinant growth factor drug is a glycemic regulatory factor, insulin, developed by Genentech in 1982.^[^ ^5^ ^]^Since then, growth factor replacement therapy using recombinant proteins or enzymes, or other similar surrogates, has experienced rapid growth and development. For the last three decades, growth factors have been used as replacement therapy for a number of deficiency syndromes, including those associated with hematologic, oncologic, and cardiovascular diseases.^[^ ^6^, ^7^, ^8^ ^]^

Recombinant growth factors, in spite of being at center stage in current replacement therapy, encounter significant challenges when used chronically in the clinical setting, including short serum half‐life, low bioavailability, dose‐limiting toxicity, and immunogenicity.^[^ ^9^, ^10^ ^]^ For example, low molecular weight growth factors often require modification to extend their half‐life.^[^ ^11^, ^12^, ^13^, ^14^ ^]^ These modifications, in turn, increase their immunogenicity. Growth factors with poor bioavailability often fail to meet desired endpoints due to their hydrophobicity. Growth factors with multiple cognate receptors other than the target often require high dosage treatment leading to dose‐limiting toxicities. Last but not the least, tolerance to recombinant growth factors is often terminated over the course of treatment,^[^ ^15^ ^]^ leading to an immune response against both the recombinant and natural growth factors.

Combinatorial antibodies named because of their selection from combinatorial antibody libraries,^[^ ^16^, ^17^ ^]^ on the other hand, have been successful in clinical intervention in a wide variety of diseases, including cancer, chronic inflammatory diseases, autoimmunity, and metabolic syndromes.^[^ ^18^, ^19^ ^]^ As one of the fastest growing therapeutics, and taking advantage of the infinite diversity of combinatorial antibodies, various innovative strategies and applications have been developed targeting challenging pharmaceutical targets such as G Protein‐Coupled Receptor (GPCRs), ion‐channels, etc.^[^ ^20^, ^21^, ^22^, ^23^ ^]^ In addition to conventional neutralization, combinatorial antibodies have shown mechanism‐based modulation of various signaling pathways,^[^ ^16^, ^17^, ^24^ ^]^ and in some cases, function beyond the scope of native ligands.^[^ ^25^, ^26^, ^27^ ^]^ As they are themselves immunoglobulins, loss of immune‐tolerance would be less of a concern for chronic administration of antibodies.

Leptin, a key physiological modulator of energy balance, was first discovered in 1994 by Friedman and co‐workers using a positional cloning strategy.^[^ ^28^ ^]^ Leptin is released into the bloodstream by white adipose tissue at a level proportional to body fat. After entering the brain it activates sets of hypothalamic neurons expressing the leptin receptor (LepR), which results in inhibition of food intake and stimulation of energy expenditure.^[^ ^29^, ^30^, ^31^, ^32^ ^]^ The leptin receptor is a member of the class I cytokine receptor family, and shares structural homology with many important hematopoietic factor receptors, such as glycoprotein 130, leukemia inhibitory factor receptor, oncostatin M receptor, ciliary neurotrophic factor receptor, and others.^[^ ^33^, ^34^, ^35^ ^]^ Its downstream signaling pathway, transduced via Janus kinase (JAK) and signal‐transducer‐and‐activator of transcription (STAT) factor, regulates subsequent kinase activities including PI3K, MAPK, ERK, and AMPK.^[^ ^36^, ^37^, ^38^ ^]^ Beyond its central role in energy homeostasis and body weight control, leptin is also a pleiotropic cytokine involved in reproduction, immune system development, bone formation, and even tumorigenesis.^[^ ^39^, ^40^, ^41^, ^42^ ^]^ Deficiency or dysfunction in leptin and/or its receptor leads to abnormal hyperphagia, excessive fat accumulation, and other related severe metabolic disorders.^[^ ^43^, ^44^ ^]^ Leptin deficiency can be triggered by many factors, such as congenital leptin gene mutation, highly active antiretroviral therapy in human immunodeficiency virus (HIV)‐infected patients, and multiple metabolic derangements with deficient adipose mass.^[^ ^45^, ^46^, ^47^ ^]^ In 2013, the FDA approved a recombinant analog of human leptin, Metreleptin, for the treatment of congenital or acquired generalized lipodystrophy.^[^ ^48^ ^]^

In the current study, we used an integrated approach combining both function‐guided cellular screening and optimization, as well as affinity maturation, to generate an antibody with the full agonist activity of leptin from a combinatorial antibody library containing 10^11^ members.^[^ ^22^, ^49^ ^]^ We designed and constructed two independent cellular assays that are suitable for intracellular combinatorial library screening^[^ ^25^, ^50^, ^51^, ^52^ ^]^ and optimization. After stepwise reiterations of function and affinity, we identified a combinatorial antibody (H6) showing functional profiles identical to leptin both in vitro and in vivo. Like leptin, H6 showed high potency and maximal efficacy across species (human and murine), and rescued leptin‐deficiency syndrome in ob/ob mice. Furthermore, as expected, H6 showed long lasting biostability in vivo, and remarkably, activated mutant leptin receptors that usually exhibit impaired or nonresponsive downstream signaling to leptin. Thus, this combinatorial antibody represents a promising therapeutic candidate, and the selection strategy is applicable to various different cellular targets.

## 2 Results

### 2.1 Combinatorial Enrichment and Optimization of Agonist Antibodies Targeting the Leptin Receptor

**Figure 1:**
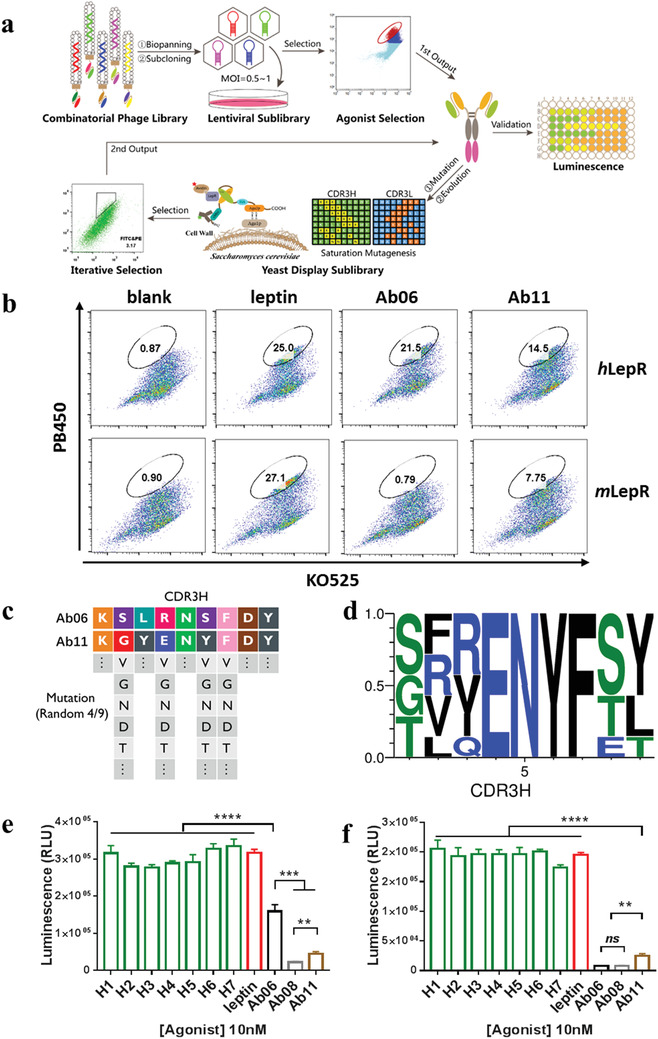
Selection of agonist antibody for leptin receptor (LepR). a) Schematic illustration for selection of agonist antibody ligands for leptin receptor (LepR). b) The purified single‐chain antibodies Ab06 and Ab11 recognize and activate LepR in *β*‐lactamase reporter cells. Upon activation of LepR, *β*‐lactamase is recruited, and followed by intracellular cleavage of CCF4‐AM (Invitrogen) substrate resulting in emission wavelength shift from green (520 nm, KO525) to blue fluorescence (450 nm, PB450). c) Sequence alignment of CDR3H (complementary determining region 3, heavy chain) of Ab06 and Ab11, and illustration of construction of a 2 × 10^7^ site‐saturation mutagenesis antibody sublibrary by random selection of four amine acid residues followed by saturated site‐mutagenesis at each residue in CDR3H. d) Amino acid sequence comparisons of CDR3H of optimized antibody candidates with high affinity and agonist activity. The selected antibodies displayed a signature enrichment pattern. e) Characterization of agonist activities against *h*LepR in luciferase reporter cell lines. Each bar represents the average of the triplicates of LepR activation at 10 × 10^−9^ m ligands. f) Characterization of agonist activities against *m*LepR in luciferase reporter cell lines. Each bar represents the average of the triplicates of LepR activation at 10 × 10^−9^ m ligands. All of the data are shown as means ± SEM (error bars), ns, not significant, ^**^ *p* < 0.01, ^***^ *p* < 0.001, ^****^ *p* < 0.0001. Statistical analyses were performed using unpaired t‐test.

We used three consecutive enrichment steps, each taking into account the features of leptin in binding and signaling with its cognate receptor, LepR, to select an agonist antibody. As shown in **Figure** **1**a, we first used affinity panning against the extracellular domain (ECD) of LepR to select ≈10^6^ binding antibodies from a naïve phage in‐put library containing 10^11^ members. After two rounds of enrichment, the focused 10^6^ sublibrary was packaged using lentivirus, and displayed on cell membranes for intracellular function‐based autocrine selection. This step was followed by affinity maturation using yeast display to improve the binding affinity of the selected combinatorial antibodies.

Leptin has been shown to activate LepR by cross‐interacting with inactive predimerized LepRs to form a 2:2 active signaling complex,^[^ ^33^, ^34^, ^53^ ^]^ of which, at least two plausible binding modes, stepwise and concerted, could be in operation. Therefore, we constructed a recombinant ECD of human LepR (*h*LepR) with a C‐terminal 6×His tag or human‐Fc tag to prepare monomeric or dimeric panning antigens, respectively. In order to identify antibodies that duplicate leptin's activation mechanism, during affinity panning, in addition to glycine–HCl (pH 2.0), we used a high concentration of leptin to competitively elute ECD‐bound phage particles (Table S1, Supporting Information). After two rounds of enrichment, the focused 10^6^ sublibrary was transferred to a lentiviral vector, and expressed in a membrane‐tethered format on the surface of a *h*LepR reporter cell line as previously described.^[^ ^25^ ^]^ The reporter cell line contains a SIS‐inducible element (SIE) responsive to STAT3 phosphorylation. In spite of a longer incubation process, the reporter gene detection using *β*‐lactamase activation showed the best signal to noise ratio and was chosen for the autocrine selection. Whereas the luciferase or GFP detection, because of its quick response, was used for hit validation (Figure S1a, Supporting Information).

It is interesting to note that only the sublibrary eluted with glycine–HCl (pH 2.0) from the dimeric ECD of *h*LepR contained agonists. The agonist antibodies appeared to bind an epitope sequence on *h*LepR different from leptin. Two single‐chain combinatorial antibodies (scFv), Ab06 and Ab11, were selected at high frequency as determined by sequence analysis, and shown to be weakly active in the *h*LepR reporter gene assays (Figure 1b,e; Figure S1c, Supporting Information). Converting the combinatorial antibodies from scFv to full‐length IgG1 abolished the agonist activity, but not the binding to membrane *h*LepR as demonstrated by immunostaining (Figure S1d, Supporting Information). Thus, unlike the scFv antibodies, the interaction between full‐length antibodies and membrane *h*LepR appeared to interfere with the homodimerization of membrane *h*LepR. Of the two agonist scFv antibodies, Ab11 showed cross‐species affinity, and could activate both human and mouse LepR signaling similarly to leptin. In contrast, Ab06 recognized and activated only *h*LepR (Figure 1b,e). Sequence alignment of the CDR regions of Ab06 and Ab11 indicated that the heavy chain CDR3 region (CDR3H) is, as expected, the most diversified region. The two hit scFv antibodies, Ab11 and Ab06, showed identical sequences except for the four residues in the CDRH3 region (Figure 1c; Figure S1c, Supporting Information), suggesting that CDR3H is the decisive region for the species specific recognition of LepR.

In order to select antibody ligands with leptin‐like, cross‐species full agonist activity, we carried out affinity maturation using yeast display by introducing site‐saturation mutagenesis into the CDR3H region of Ab11. We randomly selected four out of the nine amino acids from the CDR3H region of Ab11 as positions for saturated mutagenesis, and generated a combinatorial library containing 2.0 × 10^7^ diversified antibody sequences that, in theory, should include both Ab06 and Ab11 sequences (Figure 1c). Using yeast display and cell sorting according to the binding signal of fluorescent‐labeled ECD from human or mouse LepR (Figure S2, Supporting Information), after four rounds of enrichment against *m*LepR‐ECD followed by two rounds against *h*LepR‐ECD, seven antibody sequences (H1–H7) were isolated. A common motif, ENYF, in the CDR3H region was revealed by sequence alignment (Figure 1d). As expected, the same motif also exists in the initial agonist antibody that recognized both *h*LepR and *m*LepR, Ab11. All seven new combinatorial antibodies showed enhanced binding affinity and agonist activity for both *h*LepR and *m*LepR (Figure 1d, Figure S3, Supporting Information). Ab08 is an isotype control antibody without agonist activity from second round phage panning. We chose the H6 combinatorial antibody for further study because of its optimal stability and high expression yield (Figure S3c, Supporting Information).

### 2.2 Cellular Functions of Combinatorial Antibody H6

**Figure 2:**
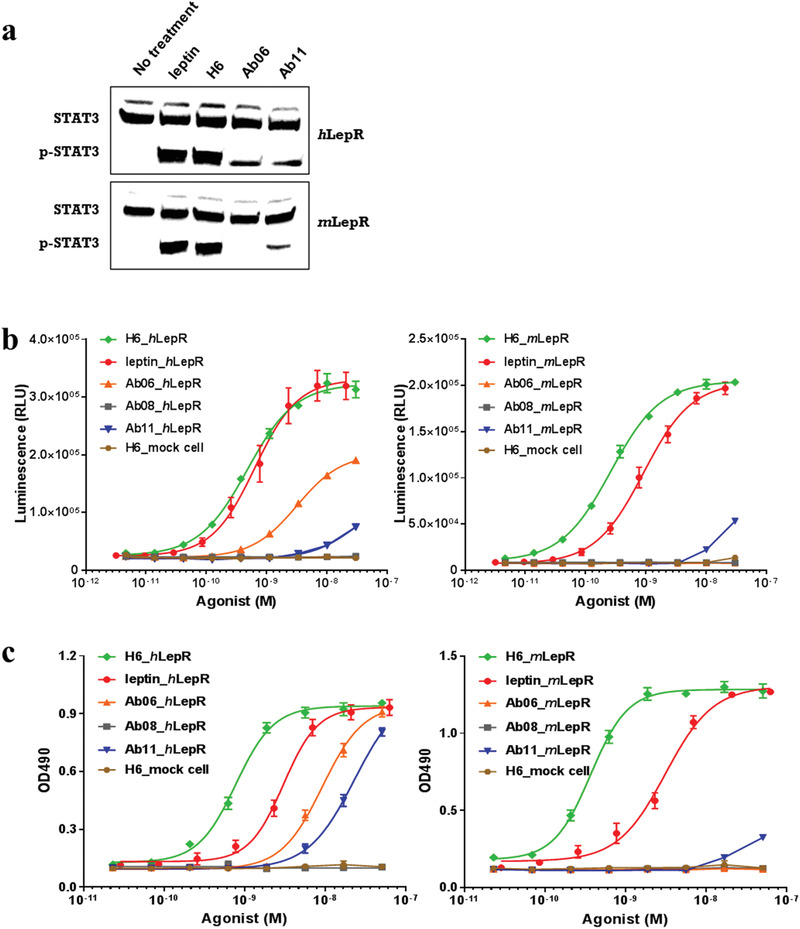
Cellular functions of the combinatorial antibody ligands. a) Western‐blot of STAT3 and phospho‐STAT3 in LepR‐expressing HEK293 cells treated with/without 10 × 10^−9^ m leptin, H6, Ab06, and Ab11, respectively. b) Dose‐dependent activation of LepR by leptin and combinatorial antibody ligands Ab06, Ab08, Ab11, and H6 via chemiluminescent detection (luminescence) of STAT3 phosphorylation in LepR or non‐LepR expressed luciferase reporter cell lines. Data are from three independent experiments and shown as means ± SEM (error bars). c) Dose‐dependent activation of LepR by leptin and combinatorial antibody ligands Ab06, Ab08, Ab11, and H6 via leptin‐dependent cell proliferation of murine IL‐3‐dependent LepR expressed or non‐LepR expressed Ba/F3 pre‐B cells (OD490, absorption at wavelength 490 nm). Data are from three independent experiments and shown as means ± SEM (error bars).

In order to compare the agonist function of H6 with that of leptin, we developed two cellular assays to measure leptin receptor‐mediated functions, namely, STAT3 phosphorylation and leptin‐dependent cellular proliferation. STAT3 phosphorylation was determined using western blot staining or chemiluminescent detection of the downstream signal generated by SIE coupling with LepR in HEK293 cells. Leptin‐dependent proliferation was measured using a murine IL‐3 dependent Ba/F3 pre‐B cell that overexpresses LepR. Western blot analyses showed that H6 and leptin had comparable activation of LepR‐mediated STAT3 phosphorylation. Whereas Ab06 and Ab11 were significantly less active (**Figure** **2**a). As expected, Ab06 showed no activation of *m*LepR (Figure 2a). The EC_50_ values for leptin and antibody ligands were determined using the chemiluminescent detection of STAT3 phosphorylation in HEK293 cells or leptin‐dependent cellular proliferation assays in engineered pre‐B cells (**Table** **1**). Ab06 and Ab11 showed only partial agonist activity and weaker potency compared to H6 and leptin (Figure 2b,c), consistent with the results of the western blot analyses. In contrast, when compared to leptin, H6 showed comparable efficacy (Figure 2b,c), and similar or more potent activation in both functional assays (Table 1). Notably, while the H6 antibody showed similar efficacy and potency in both functional assays, the natural ligand, leptin, was approximately tenfold less potent in the cellular proliferation assay compared to the phosphorylation STAT3 reporter assay (Table 1). The leptin‐dependent cellular proliferation assay requires a 72 h incubation time, while the phosphorylation STAT3 reporter assay requires only a 6 h incubation. Leptin's reduced potency with longer incubation time may reflect its biostability in cell culture.

**Table 1:** Summary of EC_50_ values of leptin and the scFv antibody ligands. Antibodies and leptin in the table represent the corresponding scFv‐Fc and leptin‐Fc format, respectively. Data are from three independent experiments and shown as means ± SEM. N/A and N/D represent not applicable and not determined, respectively

ASSAY/EC_50_ [nm]	H6	Ab06	Ab11	Leptin
*h*LepR‐luciferase	0.48 ± 0.036	3.2 ± 0.51	N/D	0.64 ± 0.069
*m*LepR‐luciferase	0.26 ± 0.011	N/A	N/D	0.91 ± 0.058
*h*LepR‐cell proliferation	0.77 ± 0.044	9.1 ± 0.62	23 ± 2.8	3.0 ± 0.20
*m*LepR‐cell proliferation	0.37 ± 0.019	N/A	N/D	3.1 ± 0.30

### 2.3 Biochemical Characterization of Combinatorial Antibody H6

**Figure 3:**
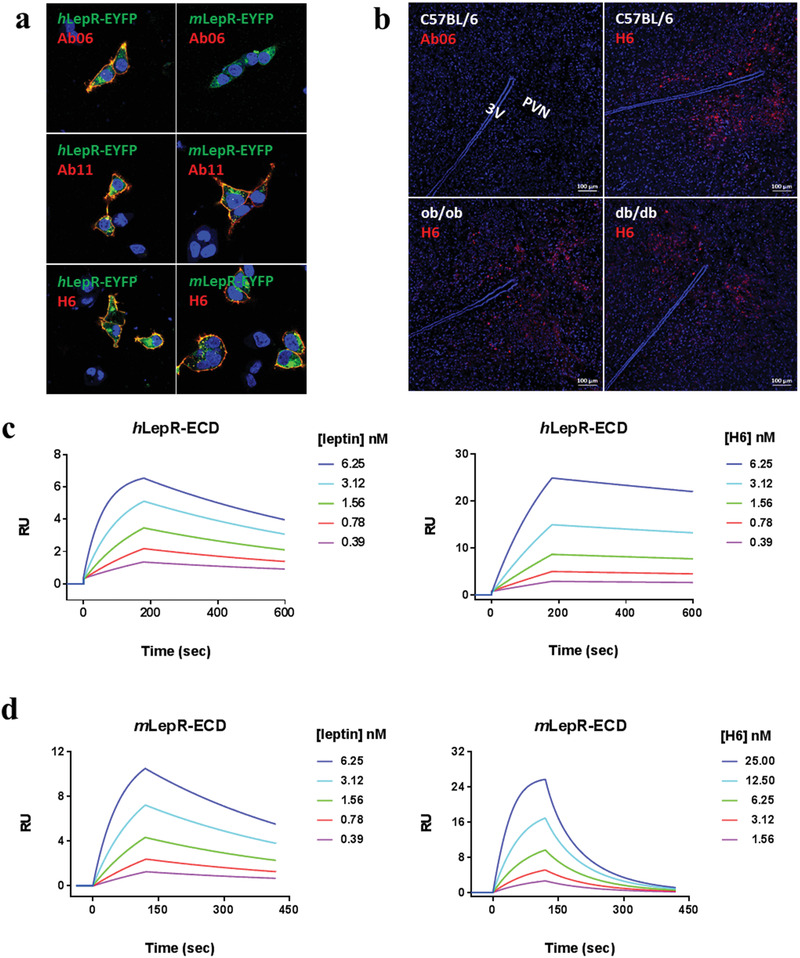
Characterization of specificity and binding affinity of combinatorial antibody ligands. a) Immunofluorescence staining of antibody ligands Ab06, Ab11, and H6 against transient transfected HEK293 cell expressing *h*LepR‐eYFP or *m*LepR‐eYFP. Cells without green immunofluorescence serve as mock control. b) Antibody ligand staining of brain tissue sections of wide type, ob/ob, and db/db mice, respectively. As is shown in the hypothalamus region, H6 but not Ab06, was co‐localized with *m*LepR in the paraventricular nucleus (PVN). 3 V, third ventricle, is marked. c,d) Comparison of binding affinities of leptin and H6 to *h*LepR‐ECD and *m*LepR‐ECD, respectively. SPR detection and five different concentrations of leptin and H6 were used.

To determine the binding specificity of H6 to the LepR, we carried out immuno‐fluorescence staining using both stable cell lines overexpressing LepR and mouse brain tissue sections. As shown in **Figure** **3**a, H6 and Ab11 recognized both *h*LepR and *m*LepR, whereas Ab06 recognized only *h*LepR in the stable cell lines expressing the corresponding receptors. These results are consistent with the functional studies noted above.

Specific recognition of LepR in tissue was further confirmed using mouse brain tissue sections extracted from wild type (WT), leptin knock‐out (ob/ob), and LepR intracellular domain partial knock‐out (db/db) C57BL/6 mice. All these mice are known to express an intact ectodomain (ECD) of LepR. As expected, H6 but not Ab06 was able to stain the region of paraventricular nucleus (PVN) in brain hypothalamus sections that consists of highly expressed *m*LepR from all three types of mice (Figure 3b).

We next measured the binding affinity of natural and antibody ligands to the ECDs of *h*LepR and *m*LepR using surface plasmon resonance (SPR). The initial antibody hits, Ab06 and Ab11, showed lower binding affinity to the ECDs of both *h*‐ and *m*LepR, consistent with the functional results (Table 1). As shown in **Table** **2**, the kinetic parameters, i.e., on‐rate (*k*_on_), off‐rate (*k*_off_), and binding constant (*K*_D_) of leptin were similar for *h*‐ and *m*LepR. On the other hand, when compared to leptin, H6 showed comparable binding affinity to the extracellular domain of *h*LepR, but two orders of magnitude less binding affinity to *m*LepR, which can be largely attributed to its greatly elevated off‐rate for *m*LepR (Figure 3c,d). Thus, H6 appeared to activate *h*LepR and *m*LepR similarly despite the greatly reduced binding affinity to *m*LepR. Diverse ligands with different binding affinities eliciting similar signaling responses have also been reported for other cytokines, such as the type I IFN family and IL‐13 variants.^[^ ^54^, ^55^ ^]^ The above characteristics of antibody H6 can be reconciled with the notion that receptor activation is a complex and dynamic process that includes not only ligand binding, but also receptor–ligand complex assembly, reconstitution, phosphorylation, and sometimes endocytosis.

**Table 2:** Summary of kinetic constants of leptin and scFv antibody ligands in binding affinity measurement with LepR‐ECD. Data are displayed using scientific notation. Antibodies and leptin in the table represent the corresponding scFv‐Fc and leptin‐Fc format, respectively. Data were processed by BIA evaluation software and *K*_D_ values were calculated by fitting the curve in 1:1 binding model. All kinetic parameters (*K*_D_, *k*_on_, and *k*_off_) are calculated by the average of five different ligand concentrations. N/A represents not applicable

Ligands	*h*LepR‐ECD			*m*LepR‐ECD		
	*K*_D_ [M]	*k*_on_ [1 M^−1^ s^−1^]	*k*_off_ [1 s^−1^]	*K*_D_ [M]	*K*_on_ [1 M^−1^ s^−1^]	*k*_off_ [1 s^−1^]
Leptin	5.0 × 10^−10^	2.6 × 10^6^	1.3 × 10^−3^	7.9 × 10^−10^	2.8 × 10^6^	2.2 × 10^−3^
H6	3.9 × 10^−10^	7.9 × 10^5^	3.1 × 10^−4^	1.7 × 10^−8^	2.1 × 10^6^	3.5 × 10^−2^
Ab06	7.5 × 10^−9^	6.9 × 10^6^	5.2 × 10^−2^	N/A	N/A	N/A
Ab11	2.4 × 10^−8^	4.7 × 10^5^	1.1 × 10^−2^	2.0 × 10^−7^	9.6 × 10^4^	1.9 × 10^−2^

### 2.4 Binding of H6 versus Leptin on LepR‐ECD

**Figure 4:**
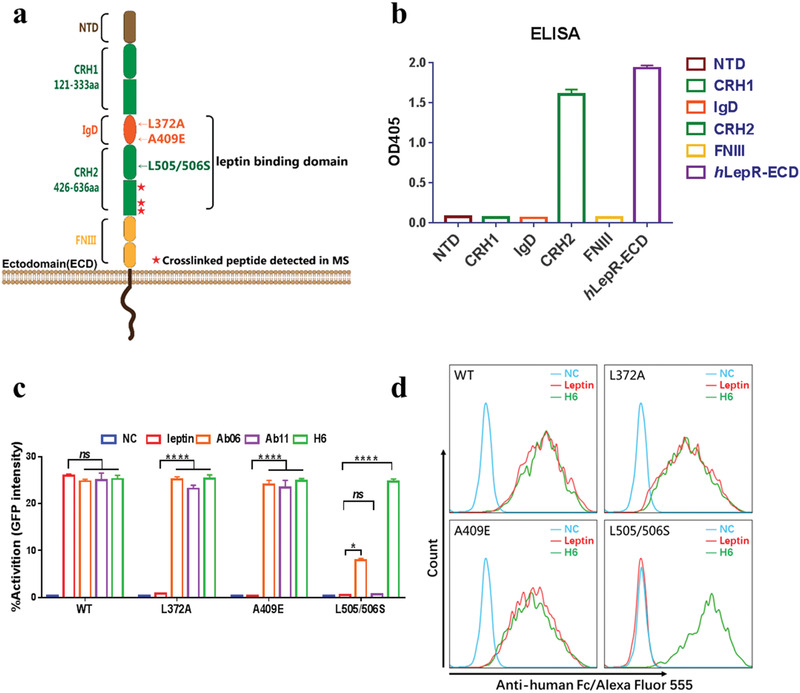
Comparison of epitope regions of LepR for leptin and H6. a) Schematic representation of LepR ectodomain (LepR‐ECD). As is shown, LepR‐ECD consists of five motifs based on their functions, N‐terminal domain (NTD), cytokine receptor homology domain 1 (CRH1), immunoglobulin‐like domain (IgD), cytokine receptor homology domain 2 (CRH2) and membrane‐proximal fibronectin type III domain (FNIII). Arrows show the different crucial mutations for disrupted leptin binding or downstream signaling. Stars represent the antibody ligand‐*h*LepR complex crosslinked peptide detected by Mass Spectrum analysis. b) ELISA analysis of H6 binding to *h*LepR‐ECD and five recombinant extracellular domains of *h*LepR. Data are from three independent experiments and shown as means ± SEM (error bars). c) Percentage activation of leptin and antibody ligands (Ab06, Ab11, and H6) to *h*LepR assessed by GFP expression generated by SIE coupling with *h*LepR in HEK293 cells. NC represents an isotype antibody ligand. Data are from three independent experiments and shown as means ± SEM (error bars), in which ns represents not significant, ^*^ *p* < 0.05, ^****^ *p* < 0.0001. Statistical analyses were performed using unpaired t‐test. d) Binding of H6 (green traces) and leptin‐Fc fusion protein (red traces) to cells overexpressing wild type (WT) and mutant (L372A, A409E, and L505/506S) LepRs was analyzed by flow‐cytometry. Blue traces represent the nonbinding control of an isotype antibody to cells overexpressing wild type and mutant LepR.

The ectodomain of LepR (LepR‐ECD) has been shown to consist of a N‐terminal domain (NTD), a cytokine receptor homology domain 1 (CRH1), an immunoglobulin‐like domain (IgD), a cytokine receptor homology domain 2 (CRH2) that is also called leptin binding domain (LBD), and two membrane‐proximal fibronectin type III domains (FNIII) (**Figure** **4**a, Table S2, Supporting Information).^[^ ^34^, ^53^, ^56^ ^]^ We used a chemical crosslinking experiment with disuccinimidyl sulfoxide (DSSO) to map the regions of interaction between H6 and *h*LepR. Three crosslinked peptides on *h*LepR‐ECD, AVQVRC[K]RL, DA[K]SKSVSLPVPDLCAVY, and E[K]PVFPENNLQF, were identified by tandem MS spectrometry analyses,^[^ ^57^ ^]^ which are located within the CRH2. To validate that no ECD regions other than CRH2 interact with H6, we overexpressed each specific domain in *h*LepR‐ECD individually (Figure 4a) and assessed its binding capability to H6 using an ELISA assay. As shown in Figure 4b, binding of H6 was only observed with *h*LepR‐ECD or the CRH2 domain consistent with the crosslinking results. To investigate whether the scFv antibody ligands and leptin are mutually exclusive, a competition assay was performed using ELISA. Leptin was first immobilized on a microtiter plate, and then mixed and incubated with a mixture of 20 × 10^−9^ m *h*LepR‐ECD‐Fc and serial diluted antibody ligands or leptin. The microtiter plate was thoroughly washed to remove the ligand‐bound *h*LepR‐ECD‐Fc. The *h*LepR‐ECD‐Fc captured by immobilized leptin was visualized and quantitated by ELISA reading at OD405. As shown in Figure S4 in the Supporting Information, all three antibody ligands (H6, Ab06, and Ab11) displayed a dose‐dependent competition with leptin for *h*LepR‐ECD‐Fc as expected.

Many pathogenic mutations in the ectodomain of LepR have been identified from severe early‐onset obesity patients.^[^ ^58^, ^59^ ^]^ Several key mutation sites that could disrupt or attenuate the LepR signaling were also reported by structure analysis.^[^ ^60^ ^]^ These mutations (Figure 4a) resulted in either loss of leptin binding or LepR desensitization. X‐ray crystallography and negative staining electron microscopy have been used to study the leptin binding site on LepR via complex structures of leptin‐LepR.^[^ ^34^, ^53^, ^56^ ^]^ We assessed the recognition and activation of wild type and mutant *h*LepR by leptin and H6 using a modified reporter gene assay, in which GFP expression is used as the downstream signal generated by SIE coupling with LepR in HEK293 cells. To determine the binding capability of H6 and leptin, recombinant proteins with human‐Fc tags were constructed and purified for both H6 and leptin to be used in immunofluorescence staining analyses. As shown in Figure 4c,d, for the two *h*LepR mutants, L372A and A409E, which have been identified as monogenic causes of early‐onset obesity due to defective leptin signaling, H6 could readily interact and activate both of these mutant receptors. In contrast, as previously reported in the literature, leptin showed only binding to but no activation of these mutant receptors. When tested with the *h*LepR mutant containing a L505/506S double mutation that is known to disrupt the interaction between *h*LepR and leptin, resulting in loss of function of leptin signaling, again H6 showed effective binding and activation (Figure 4c,d). H6 likely binds to the same CRH2 region as leptin, but has different contact residues. Thus, this combinatorial antibody represents a new class of agonist ligands for LepR signaling, and a potential therapeutic for that class of patients who show leptin deficiency or resistance as a result of receptor mutation.

### 2.5 Rescue of Leptin Deficiency in ob/ob Mice

**Figure 5:**
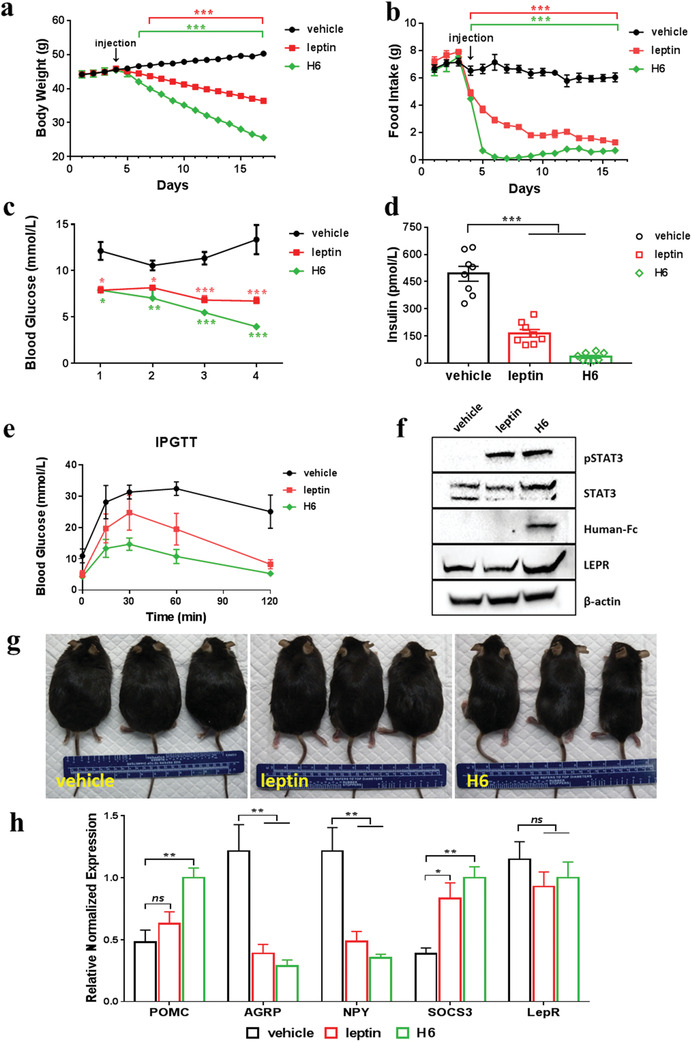
Rescue of leptin deficiency in ob/ob mice. a,b) Body weight and food intake are recorded daily for H6 group (*n* = 8, 5.0 mg kg^−1^, qod), leptin group (*n* = 8, 0.5 mg kg^−1^, bid), and vehicle group (*n* = 8, 5.0 mL kg^−1^, bid) before and during the treatment period as indicated. c) Nonfasting blood glucose was measured twice a week during treatment. d) Fasting plasma insulin concentration was measured after two weeks treatment and 16 h starvation. e) Intraperitoneal glucose tolerance test (IPGTT) was carried out after two weeks of treatment. After the glucose load (2 g kg^−1^), blood glucose levels were measured at several indicated time points. f) Hypothalamic tissue homogenate western blot. In the leptin and antibody H6 groups, phosphorylated STAT3 was detected, while there was no phosphorylation in the vehicle control group. Human Fc fragment (Human‐Fc) was also detected using a related secondary antibody, confirming that LepR was accessible to the H6 antibody in the circulation. g) Representative photos of three mice from each treatment group are shown. h) Quantification of gene expression with qRT‐PCR. Anorexigenic effect related gene, Proopiomelanocortin (POMC), agouti‐related peptide (AGRP), neuropeptide Y (NPY) are measured, as well as LepR and the negative feedback regulator, SOCS3. All mRNA levels were normalized to *β*‐actin. All of the data are shown as means ± SEM (error bars, *n* = 8), in which ns represents not significant, ^*^ *p* < 0.05, ^**^ *p* < 0.01, ^***^ *p* < 0.001. Statistical analyses were performed using analysis of variance (ANOVA).

We used a leptin‐deficient ob/ob mouse model to evaluate the in vivo efficacy of the agonist antibody, H6. The ob/ob mice, due to insufficient plasma leptin levels, display a phenotype of hyperphagia and extreme obesity accompanied by hyperglycemia and hyperinsulinemia. We carried out a quick pharmacokinetic analysis of leptin and H6 in mice before the animal model study. Using i.v. injection, five wild type C57BL/6 mice per group were used in the comparison of the plasma concentrations of leptin and H6. After normalizing the plasma concentrations of ligands to OD490 based on Ba/F3 reporter cell proliferation assay, the estimated *t*_1/2_ in plasma is less than 1 h for leptin (consistent with the literature report), whereas greater than 8 h for H6 (Figure S5a, Supporting Information). The subcutaneous injection dosages of H6 (5 mg kg^−1^) and leptin (0.5 mg kg^−1^), which are roughly equivalent to 1 × 10^−6^ and 0.8 × 10^−6^ m, respectively, and 1000 times higher than the efficacious concentrations in vitro, were thus selected to assure the circulating leptin or H6 is sufficient to elicit a beneficial response. In general, H6 (5 mg kg^−1^, qod), leptin (0.5 mg kg^−1^, bid), or vehicle (5 mL kg^−1^, bid) were injected subcutaneously into three groups of ob/ob mice (eight mice per group) for two weeks. During treatment, these mice were monitored daily for body‐weight and food‐intake. Both the leptin‐treated and antibody‐treated group showed significant and sustained decrease in body weight and food intake, while the vehicle control group showed no significant change in food intake and the expected gradual increase in body weight (**Figure** **5**a,b). Interestingly, mice treated with the H6 antibody showed an immediate and almost complete inhibition of food intake leading to a sharp loss of body weight. In addition, unlike in the vehicle control group, blood glucose levels in both leptin‐ and H6‐treated mice decreased significantly (Figure 5c). Two weeks of treatment with leptin or H6 significantly reduced both nonfasting blood glucose and basal plasma insulin levels in these mice (Figure 5d,e). In intraperitoneal glucose tolerance tests (IPGTT), 30 min after the i.p. glucose injection (2 g kg^−1^), blood glucose concentrations increased in both treated and control mice. However, glucose levels remained significantly lower in leptin‐ and H6‐treated mice throughout the experiment (120 min) (Figure 5e). In summary, in the three parameters tested (weight, food consumption, glucose tolerance) treatment of ob/ob mice with antibody H6 resulted in a normalizing effect that was even greater than that seen with leptin itself. In spite of significantly less food intake, all animals appeared active and healthy. As expected, mice treated with either H6 or leptin were visibly leaner (Figure 5g), and whole body adipose tissue and liver weights were decreased significantly (Figure S5, Supporting Information). The presence of H6 antibody in hypothalamic tissue was confirmed using immunoblotting against the human antibody Fc fragment (Figure 5f).

The anorexigenic effect of leptin has been shown to involve LepR‐dependent transcriptional regulation of hypothalamic neurons expressing proopiomelanocortin (POMC), agouti‐related peptide (AGRP), and neuropeptide Y (NPY).^[^ ^30^, ^37^ ^]^ To determine if treatment with H6 has downstream signaling effects similar to leptin, we collected hypothalamic tissue from treated mice and assessed the mRNA level of these genes, as well as suppressor of cytokine signaling (3SOCS3), a negative feedback regulator of leptin signaling.^[^ ^36^, ^61^ ^]^ As shown in Figure 5h, treatment with leptin and H6 showed similar mRNA expression profiles for all the genes examined. Moreover, the mRNA expression level of *m*LepR remained unchanged before and after treatment.

To rule out other nonspecific activities that may associate with the combinatorial antibody, we tested antibody H6 in both WT and intracellular domain of *m*LepR knock‐out db/db mice models. Similar to leptin, after two weeks of treatment, H6 showed no significant impact on either body‐weight or food‐intake in db/db mice. However, as a result of significantly reduced food intake, H6‐treated WT mice showed a steady decrease in body weight (Figure S6, Supporting Information).

## 3 Conclusion

In the work reported here, we took advantage of a combinatorial antibody library with 10^11^ members and the full panoply of modern immunochemical methodology to develop a highly efficient reiterative panning/functional selection and optimization (both affinity and function) process targeting both *h*LepR and *m*LepR. Using these methodologies we successfully selected an agonist antibody (H6) with characteristics identical to the natural ligand, leptin, both in vitro and in vivo. Key to success was starting with very large numbers, followed by maximum randomization at each iteration to achieve optimal diversity. For example, during the affinity maturation process we used site‐saturation mutagenesis of four randomly selected amino acids in the CDR3H region of the initial antibody hit (Ab11). The resulting combinatorial antibody ligand (H6) binds to LepR with the same affinity and in the same CHR2 region as leptin, but likely utilizes different contact residues and follows a different binding mechanism. Leptin is believed to bind to LepR by cross‐interacting with inactive predimerized LepRs to form a 2:2 active signaling complex,^[^ ^33^, ^34^, ^53^ ^]^ in which leptin has two cognate binding sites (primary and secondary) located on two separated regions of LepR. Leptin cross‐interacts with the two sites on two LepRs. The primary interaction between leptin and LepR brings two LepRs closer to form an inactive predimer complex. Activation occurs upon the secondary interactions and the formation of a 2:2 active homodimer complex. The homodimerization of LepR appeared to be the key for LepR mediated signaling. The difference in the LepR activation between scFv and IgG (and Fab) is most likely due to the geometry of binding domains in the scaffold of a full length (or Fab) antibody. Symmetry in a ligand is, thus, important to a 1:2 functional complex. This unique feature allowed the scFv antibody ligand to overcome two examples of LepR pathogenic mutations related to leptin insensitivity and resistance (L372A and A409E), making it superior to the natural ligand in some therapeutic situations. In addition, H6 displays the usual immunoglobulin characteristics of desirable bioavailability and stability in vivo.

As antibodies have valuable therapeutic advantages in the treatment of various diseases, many agonist antibodies have been generated using a variety of screening methods. However, most of them are not full agonist antibodies, and show lower potency than the natural ligands thereby limiting their further utility.^[^ ^62^, ^63^ ^]^ The specificity of antibody binding can also result in some agonist antibodies only being able to recognize the receptor from one species, having lost cross‐species activation capability. As previous studies reported, two agonist antibodies targeting the leptin receptor generated from different selection methods exhibit potent activity against either mouse or human leptin receptor.^[^ ^64^, ^65^ ^]^ Our function‐guided selection approach and optimization process for full agonist antibody generation solves such issues.

Usually, if an antibody works, so will a small molecule targeting the same region. Thus, this study can be considered as a prelude to the selection of a small molecule ligand using DNA‐encoded library technology.^[^ ^66^ ^]^ One would again start with a very large library, in this case of organic molecules, and competitively elute the binders using leptin. Small organic molecules may have some additional therapeutic advantages, such as oral availability, and most importantly, synergy with leptin.

## 4 Experimental Section

##### Cell Culture

HEK293 cell line was maintained in high glucose Dulbecco Modified Eagle Medium (DMEM) (Gibco) containing 10% (v/v) fetal bovine serum (FBS) (Gemini), the FreeStyle 293‐F cell line (Gibco) was cultured in Freestyle 293 expression media (Gibco), the murine IL‐3‐dependent cell line Ba/F3 (DSMZ) was maintained in RPMI‐1640 (Gibco) containing 10% (v/v) dialyzed fetal bovine serum (DFBS) (Gemini) and 2 ng mL^−1^ rmIL‐3 (R&D Systems). For yeast display strain EBY100, yeast cells with display vectors were cultured in SD/Trp^−^ media (Clontech) to logarithmic phase at 30 °C with shaking. For induction, yeast cells with display vectors were induced in SGRCAA media (yeast nitrogen base 6.7 g L^−1^, casamino acid 5 g L^−1^, galactose 20 g L^−1^, Raffinose 20 g L^−1^, NaH_2_PO_4_ 7.44 g L^−1^, Na_2_HPO_4_ 5.39 g L^−1^) for 24 h at 20 °C with shaking.

##### Animals

All animal experiments were approved by the Institutional Animal Care and Use Committee of ShanghaiTech University. Six to eight week old male ob/ob (C57BL/6‐Lep^em1Cd25^/Nju), db/db (BKS‐Lepr^em2Cd479^/Nju) and wild type (C57BL/6J‐Nju) mice were purchased from GemPharmatech Co., Ltd. All animals were maintained in pathogen‐free, temperature‐controlled environment with a 12 h light/dark cycle and provided chow and water ad libitum. All animals were allowed to acclimate for at least one week in the animal facility before handling. Before each experiment, body weight and food intake of mice were monitored daily for 3–4 days prior to the injections. All selected mice were randomly divided into indicated groups. During treatment, mice of each testing group (eight mice per group) were injected subcutaneously the corresponding recombinant human leptin (0.5 mg kg^−1^, twice daily), H6 (5 mg kg^−1^, once every other day), and vehicle control (Phosphate Buffer Saline (PBS), twice daily). Leptin and H6 were diluted appropriately in sterile phosphate buffered saline and administered in a dose volume of 5 mL kg^−1^. After injection, food intake and body weight of each mouse were monitored on a daily basis. Food intake was calculated from the weight difference of the daily remaining food in the cage.

##### Expression and Purification of Recombinant Proteins

The coding sequence of extracellular domain of human and mouse leptin receptor (*h*LepR‐ECD‐ and *m*LepR‐ECD) were cloned into expression vector with either 6×His tag or human‐Fc tag on C‐terminus. HEK293F overexpressing each recombinant protein was cultured for 4 days. Recombinant proteins were harvested from the supernatant. HisTrap FF Crude column (GE Healthcare) and HiTrap Protein A HP column (GE Healthcare) were used to purify his‐tagged and *h*Fc‐tag proteins, respectively. Detailed procedures were followed with manufacturer's instruction. Briefly, for His‐tagged protein, after loading of the supernatant to a HisTrap FF Crude column, the column was washed with a binding buffer (500 × 10^−3^ m NaCl, 20 × 10^−3^ m sodium phosphate, 20 × 10^−3^ m imidazole, pH = 7.4), then eluted with an elution buffer (500 × 10^−3^ m NaCl, 20 × 10^−3^ m sodium phosphate, 500 × 10^−3^ m imidazole, pH = 7.4). For combinatorial antibodies or *h*Fc‐tagged proteins, supernatant preparation is the same as that for His‐tagged protein except for using a different binding buffer (150 × 10^−3^ m NaCl, 20 × 10^−3^ m sodium phosphate, pH = 7.4) and an elution buffer (0.1 m citric acid, pH = 3.4). All the eluted protein samples were exchanged and concentrated in a PBS buffer (150 × 10^−3^ m NaCl, 20 × 10^−3^ m sodium phosphate, pH = 7.4) using Ultracel membrane with molecular weight cutoff of 30 kDa (Merck Millipore). Final concentrations were determined by BCA Assay Kit (Thermo Scientific).

##### Focused Combinatorial Antibody Library Generation for hLepR‐ECD

A combinatorial scFv antibody library (10^11^) was enriched to yield a smaller but more specific sublibrary of about 10^6^ members after two rounds phage panning against *h*LepR‐ECD. To maximize the chance to find ideal combinatorial antibodies, three panning strategies were used with different antigens or elution conditions as summarized in Table S1 in the Supporting Information. His‐tag and *h*Fc‐tag were constructed at the C‐terminus of *h*LepR‐ECD to create monomeric and dimeric antigen, respectively. Three panning experiments using phage display were carried out in parallel, in which, for panning 1, a glycine buffer (0.1 m glycine–HCl, pH = 2.0) was used to elute the phage particles binding to *h*Fc‐tagged antigen on magnetic protein G beads (New England Biolabs); for panning 2, 0.5 m leptin in PBS buffer (pH = 7.4) was used to elute the positive phage particles; for panning 3, His‐tagged protein was coated on nickel‐coated magnetic beads (Invitrogen), and 0.1 m glycine–HCl (pH = 2.0) or 500 × 10^−3^ m imidazole in PBS (pH = 7.4) was used as the elution buffer. In the case of *h*Fc‐tagged antigen, phage particles were first pre‐incubated with saturating irrelevant recombinant human Fc protein to remove phage particles that bound nonspecifically to the *h*Fc‐tag. For each round of phage panning, XL1‐blue cells (Agilent) were infected with the eluted phage particles and grown at 30 °C overnight. The resulting XL1‐blue cells were collected followed by the addition of the helper phage VCSM13 (New England Biolabs) to amplify phage for the next round of panning. After two rounds of phage panning, ≈10^6^ colonies were collected and the corresponding phagemid DNAs were extracted.

The phagemid DNAs encoding scFv antibody sequences were then digested using restriction enzyme sfiI (New England Biolabs) and cloned into a mammalian cell surface display lentivirus vector, a member‐tethered system as described previously.^[^ ^25^ ^]^ The *h*LepR‐focused combinatorial antibody lentivirus library was established in HEK293T cells by co‐transfection of the resulting lentiviral vector with packaging plasmids pCMV‐dR8.91 and pVSVg at ratio of 1:1:1. Virus containing the focused library was harvested from the supernatant at 72 h post‐transfection. Cell debris were removed by centrifugation and filtration through a 0.22 µm filter (Millipore). The viral titer was determined using Lenti‐X p24 ELISAs kit (Clontech).

##### Intracellular Autocrine Selection of Focused Combinatorial Antibody Library

A stable line containing a *β*‐lactamase reporter gene under control of the SIE, 6× GGTTCCCGTAAATGCATCA, was first established by stably integrating the gene construct into a HEK293 cell followed by selection in response to IL‐6 stimulation. The LepR gene was then cloned into a lentivirus vector under EF1*α* promoter and transfected into the stable SIE sensor cell to yield the LepR mediated SIE‐*β*‐lactamase reporter gene cell line. The clone with the best signal to noise ratio was selected by leptin stimulation, and amplified for future screening and reporter assays.

The resulting LepR reporter gene cells were infected with lentivirus containing the *h*LepR‐focused combinatorial antibody library at Multiplicity of Infection (MOI) of 0.5–1. After 8 h inoculation, cells were exchanged into a fresh media and cultured for an additional 40 h. The resulting cells were collected and incubated with LiveBLAzer‐Fluorescence Resonance Energy Transfer (FRET) substrate CCF4‐AM (Invitrogen) for 2 h in dark, washed with PBS buffer (pH = 7.4) once, and subjected to single cell‐sorting on Fluorescence Activated Cell Sorting (FACS). In the presence of *β*‐lactamase activity, the FRET substrate was cleaved resulting in disruption of FRET signals, which led to a blue‐shift of the fluorescent emission signal from wavelength 520 nm (green) to 450 nm (blue). Each single positive cell was collected into each well of a 96‐well cell culture plate. A total of four 96‐well plates were collected and incubated at 37 °C under 5% CO_2_ for 2 weeks. The positive cells forming single clones were allowed to reach confluence, and the corresponding antibody genes from each colony were amplified by Polymerase Chain Reaction (PCR) according to lentiviral vector sequence.

##### Combinatorial Antibody Library of Site‐Saturation Mutagenesis

Site‐saturation mutagenesis combinatorial antibody library was established in a yeast display vector pCTcon2 (Addgene) using the hit antibody Ab06 as a template and introducing a stop codon in the CDR3H region of Ab06 heavy chain to generate mutation template vector. Four amino acid residues from the nine amino acid residues in CDR3H region of Ab06 were randomly selected, and their genetic codons were mutated to NNK to generate a site‐saturation combinatorial antibody library with 2 × 10^7^ in diversity. In a typical experiment, the mutation template sequence of Ab06 was linearized by PCR amplification outside the CDR3H. The site‐saturation mutation fragments of CDR3H were obtained by PCR annealing to generate duplex DNA fragments with homologous arms of 30 bp compatible to the linearized Ab06 template. Finally, the linearized Ab06 template and mutation fragments were mixed and transduced into EBY100 electro‐competent cells by electroporesis (Gene Pulser Xcell, bio‐Rad). The resulting yeast library was passaged until OD_600_ reaching to 3–5, which were kept as frozen stocks at −80 °C.

##### Yeast Surface Display and Selection

Recombinant *h*LepR‐ECD or *m*LepR‐ECD with a C‐terminal His‐tag was first labeled with biotin using an EZ‐LINK NHS‐PEG4‐BIOTIN kit (Thermo Scientific). Excess biotin was removed using a desalting column (Thermo Scientific). Yeast cells carrying the site‐saturation antibody library were cultured and induced as described previously. The resulting yeast cells were washed once with a FACS buffer (PBS, pH = 7.4, supplemented with 2 × 10^−3^ m EDTA and 0.5% BSA), and mixed with anti‐Myc chicken IgY fraction (Invitrogen) and biotinylated LepR‐ECD at room temperature for 30 min. The expression of the antibody library was detected by the expression of the c‐Myc tag, of which Streptavidin‐R‐Phycoerythrin (Invitrogen) and AlexaFluor 488 goat anti‐chicken antibody (Invitrogen) were mixed with the above yeast cells washed twice with the FACS buffer, and incubated in dark at room temperature for 30 min. The unbound Streptavidin‐R‐Phycoerythrin and AlexaFluor 488 goat anti‐chicken antibody were removed by two washes using the FACS buffer before BD FACSAria III flow‐cytometry analyses. The procedure for yeast library selection is described in Figure S2a in the Supporting Information. Briefly, *m*LepR‐ECD was used as antigen in the initial four rounds of library panning, and for each round, the concentration of *m*LepR‐ECD was sequentially reduced from 100 × 10^−9^ to 50 × 10^−9^, 10 × 10^−9^, and finally 2 × 10^−9^ m. In the fifth round of library panning, *h*LepR‐ECD (2 × 10^−9^ m) was used, whereas in the sixth round, 200 × 10^−9^ m unbiotinylated *h*LepR‐ECD was included for kinetic competition selection. Yeast display plasmids of positive clones from the final round were extracted and sequenced.

##### STAT3‐Phosphorylation Detection by Western‐Blot

Cells were cultured in a serum‐free medium overnight, then treated with serum‐free medium containing 10 × 10^−9^ m leptin, 10 × 10^−9^ m agonist antibody at 37 °C, 5% CO_2_ for 30 min. Cells were next collected and washed once with ice‐cold PBS containing Halt protease and a phosphatase inhibitor mixture (Pierce). The resulting cells were lysed on ice for 30 min with a RIPA lysis buffer (Amresco) containing Halt protease and phosphatase inhibitor mixture. Cell debris was removed by centrifugation at 14 000 × *g* for 10 min. The amount of total STAT3 and phosphorylated STAT3 was determined by western‐blot analysis of the cell lysates supernatant using anti‐STAT3 antibody (abcam) and anti‐pSTAT3 (Tyr705) (abcam), respectively.

##### Functional Characterization of Antibody Ligands in LepR Mediated SIE‐Luciferase Reporter Gene Stable Cells

Similar to SIE‐*β*‐lactamase reporter gene stable line, the LepR mediated SIE‐luciferase reporter gene stable line was established by substituting the luciferase gene in place of the *β*‐lactamase reporter gene. In a typical assay, the SIE‐luciferase reporter gene cells were diluted to 0.4 million cells mL^−1^ and seeded into a tissue culture treated white opaque 96 plate (PerkinElmer) (50 µL per well). Antibody stocks (100 × 10^−9^ m in PBS, pH = 7.4, 50 µL per well) were 1:3 serial diluted and mixed with reporter cells individually. The resulting cell mixtures were cultured at 37 °C under 5% CO_2_ for 6 to 8 h, and followed by the addition of substrate of ONE‐Glo Luciferase (v/v 1:1) (Promega) into each assay well. The resulting luminescence intensity of each well was measured on a microplate reader (Enspire, PerkinElmer). Data at different concentrations of each ligand were collected form three independent experiments.

##### Functional Characterization of Antibody Ligands in LepR Mediated SIE‐GFP Reporter Gene Stable Cells

Similar to SIE‐*β*‐lactamase reporter gene stable line, the LepR mediated SIE‐GFP reporter gene stable line was established by substituting the GFP gene in place of the *β*‐lactamase reporter gene. Three mutations in LepR‐ECD that disrupt or attenuate LepR signaling, L372A, A409E, and L505/506S were constructed by site‐directed mutagenesis and verified by DNA sequencing. All mutant LepR constructs were transient transfected into the SIE‐GFP reporter cells. After 24 h cultivation, leptin (10 × 10^−9^ m) and agonist antibody ligands (10 × 10^−9^ m) were added to the cells and cultivated for additional 8 h at 37 °C, 5% CO_2_. LepR mediated GFP expression was analyzed on a Cytoflex S flow cytometer. Data of each ligand were collected form three independent experiments.

##### Leptin‐Dependent Cell Proliferation Assay

A stable cell line with leptin‐dependent proliferation was constructed using an IL‐3 dependent Ba/F3 cell, in which recombinant lentivirus containing LepR gene was transfected to replace IL‐3 signaling pathway. The cell line was established by culturing in the RPMI 1640 media containing 2 ng mL^−1^ leptin. Before proliferation assay, cells were washed with PBS (pH = 7.4) three times, diluted to 0.2 million cells mL^−1^, and seeded into a 96‐well plate (50 µL per well). Stock solutions of ligands (50 × 10^−9^ m, 50 µL per well) were 1:3 serial diluted and mixed with cells. Cells were cultured at 37 °C, 5% CO_2_ for an additional 72 h. CellTiter 96 AQueous One Solution Reagent (Promega) was then added into each well of cells (20 µL per well) and incubated at 37 °C, 5% CO_2_ for 2 h. Absorbance at 490 nm of each well was recorded on a microplate reader (Enspire, PerkinElmer). Data at different concentrations of each ligand were collected from three independent experiments.

##### Competition Binding Assay

Human leptin (10 µg mL^−1^) in a PBS (pH = 7.4) buffer was incubated and coated in each well of a 96‐well microtiter plate overnight at 4 °C. After blocking by a 5% milk Phosphate Buffer Saline ‐ Tween (PBST) (0.05% tween‐20 in PBS, pH = 7.4) buffer, a mixture of 20 × 10^−9^ m *h*LepR‐ECD‐Fc with serial diluted leptin or antibody ranging from 25 × 10^−12^ to 500 × 10^−9^ m (2 h incubation at room temperature) was added at 100 µL per well into the above leptin coated plate and incubated for an additional 2 h at room temperature. The resulting plate was washed eight times with the PBST buffer. The immobilized *h*LepR‐ECD‐Fc was detected with an Horseradish Peroxidase (HRP)‐conjugated anti‐human antibody. A colorimetric reaction was produced by the 2,2'‐Azinobis [3‐ethylbenzothiazoline‐6‐sulfonic acid]‐diammonium salt (ABTS) substrate solution before measuring the absorbance at 405 nm.

##### Immunofluorescence Staining of Cell and Brain Tissue

Transient transfected HEK293 cells expressing LepR tagged with an eYFP at C‐terminus were seeded 0.2 million per well into a poly‐d‐lysine‐coated glass bottom 24‐well plates. After 24 h cultivation, cells were washed with PBS (pH = 7.4) once, fixed in 4% paraformaldehyde (PFA), and incubated for 30 min in a blocking buffer (Thermo Scientific) at room temperature. Primary antibodies (5 µg mL^−1^) were incubated with the cells at 4 °C overnight, followed by incubation with Alexa Fluor 555‐conjugated goat anti‐human IgG secondary antibody (2 µg mL^−1^, Invitrogen) for 1 h at room temperature, and finally with 4', 6‐diamidino‐2‐phenylindole (DAPI) (2 µg mL^−1^, Roche) for 15 min at room temperature. After each addition, three PBS (pH = 7.4) washes were carried out to remove extra antibodies and DAPI.

For brain tissue sections, mice were anesthetized with isoflurane and transcardially perfused with PBS (pH = 7.4) followed by 4% PFA. Brains were removed by dissection and soaked in 4% PFA overnight at 4 °C. Tissue were embedded in tissue freezing medium (Leica) and sectioned at 30 µm with freezing microtome. The sections were first washed three times with PBS (pH = 7.4), incubated in a blocking buffer (Thermo Scientific) for 30 min at room temperature, and then followed a similar staining procedure for cells as described previously. The immunofluorescence images were collected on a laser scanning confocal microscopy (ZEISS LSM710). Data acquisition and analyses were performed with the ZEN 2012 professional software.

##### SPR

Affinity binding was measured by SPR on a Biacore T200 (GE Healthcare). Recombinant LepR‐ECD with his‐tag was immobilized on the surface of Series S Sensor CM5 chip (GE Healthcare) by an Amine Coupling Kit (GE Healthcare). Stocks of leptin and different agonist antibodies (in PBS, pH = 7.4) were 1:2 serial diluted and used as analytes in liquid phase. All procedures followed the user guide of manufacturer. Data were processed by BIA evaluation software.

##### Chemical Crosslinking Mass Spectrometry (XL‐MS)

Antibody and LepR‐ECD complex was established by mixing recombinant LepR‐ECD and antibody at 1:1 ratio. Crosslinking reaction was initiated by addition of 20× excess disuccinimidyl sulfoxide (Thermo Scientific) into the above complex. After 30–60 min incubation, 1 m Tris buffer (pH = 8.0) was used to terminate the reaction. Crosslinked samples were digested by trypsin followed by desalting on a size exclusion column. LC‐MS/MS was carried out on an integrated system containing C18 precolumn (Thermo Acclaim PepMap 100, 100 µm × 2 cm) used for sample loading, C18 analytical column (Thermo PepMapTM RSLC C18 2 µm, 100A, 75 µm × 50 cm) used for sample chromatographic separation, and NanoLC‐MS/MS, EASY1000, Orbitrap Fusion (Thermo Scientific).

Peptide‐mapping data were collected on an Orbitrap Fusion Tribrid (Thermo Scientific) with an on‐line NanoLC system and analyzed using CID‐MS^2^‐MS^3^ strategy as previously described .^[^ ^67^ ^]^ Mono‐isotopic mass of parent ions and corresponding fragment ions, parent ion charge states, and ion intensities from LC MS^2^ and LC MS^3^ spectra were extracted using Xcalibur v 3.0 (Thermo Scientific). Database searching was performed using Proteome Discoverer v 2.2 software (Thermo Scientific). Chymotrypsin was set as the enzyme with two missed cleavages being allowed as the maximum values. Protein N‐terminal acetylation, methionine oxidation (15.995 Da), carbamidomethyl cysteine (57.021 Da), hydrolyzed lysine DSSO (176.014 Da), and lysine DSSO TRIS (279.078 Da) were selected as variable modifications. In addition, to account for the residual crosslinker, three defined modifications on uncleaved lysines were chosen including alkene (C_3_H_2_O, 54 Da), sulfenic acid (C_3_H_4_O_2_S, 104 Da), and thiol (C_3_H_2_SO, 86 Da) modifications. A false discovery rate (FDR) of 1% was employed to filter out false positive results. The MS, MS^2^, and MS^3^ mass tolerances were set as 10, 20 ppm, and 0.6 Da, respectively.

XlinkX detect program (Thermo Scientific) was used to search MS^2^ data and identify putative DSSO‐interlinked products based on the unique DSSO fragmentation patterns. Mono‐isotopic masses and charges of parent ions measured in MS^3^ scans for those putative crosslinked peptides were further validated and scored by XlinkX. Final results were confirmed by manual inspection of the MS^2^ and MS^3^ spectra, respectively.

##### Pharmacokinetics Studies of Leptin and Antibody

To compare the rate of plasma protein clearance of H6 and leptin, a single i.v. dosing of 35 µg H6 or 5 µg leptin in 200 µL PBS were injected into C57BL/6 wild type mice with five mice per treatment. Blood samples were collected at the following time points after injection: 1, 2, 4, and 8 h. The amounts of H6 or leptin in plasma were estimated by the Ba/F3‐*h*lepR reporter cell proliferation signal (OD490).

##### Glucose Tolerance Tests

IPGTT was carried out for mice after 16 h fasting. After measuring the basal blood glucose levels, each animal received an i.p. injection of 2 g kg^−1^ glucose. The blood glucose levels were measured at 15, 30, 60, and 120 min post glucose‐administration using a glucometer (Roche).

##### Hypothalamic mRNA Expression Level Quantification by Quantitative Real time ‐ Polymerase Chain Reaction (qRT‐PCR)

Mice were euthanized in a CO_2_ chamber, and hypothalamic tissue was collected in a test tube. Total RNA was extracted from the entire hypothalamic tissue with TRIzol Reagent (Invitrogen). cDNA was synthesized using HiScript II RT SuperMix (Vazyme). Quantitative PCR was performed with ChamQTM Universal SYBR qPCR Master Mix (Vazyme). Messenger RNA expression levels of Pomc, Agrp, Npy, Socs3, and LepR were measured and normalized to the internal control genes (*β*‐actin).

##### Statistical Analyses

All experiment results were indicated as mean plus/minus standard error (Mean ± SEM) unless stated otherwise. Data analysis was performed using regular one‐way or two‐way analysis of variance (ANOVA) for comparisons of more than two groups. When only two groups were compared, statistical significance was assessed with an unpaired Student's t‐test. All statistics were carried out with GraphPad Prism 7. Significance was considered as ^*^ *p* < 0.05; ^**^ *p* < 0.01; ^***^ *p* < 0.001; ^****^ *p* < 0.0001.

## Conflict of Interest

The authors declare no conflict of interest.

## Author Contributions

P.T., G.Y., and R.A.L. conceived the study and wrote the manuscript. P.T. designed and performed the experiments, provided materials, and analyzed the data. Y.K., Y.L., W.L., K.F., Z.G., and L.L. performed experiments. J.M.F. discussed the results and reviewed the manuscript. M.Q., Z.Z., and P.M. supervised methodological and statistical aspects of the study.

## Supporting information

Supporting Information
