# Supplementary material for: Selection of a Full Agonist Combinatorial Antibody that Rescues Leptin Deficiency In Vivo
Source: Adv Sci (Weinh). 2020 Jul 1;7(16):2000818. doi: 10.1002/advs.202000818 (PMC7435230; doi:10.1002/advs.202000818)
Supplement: Supplementary file 1 — Supporting Information [file ADVS-7-2000818-s001.pdf]

Copyright WILEY-VCH Verlag GmbH & Co. KGaA, 69469 Weinheim, Germany, 2020.

Supporting Information

Selection of A Full Agonist Combinatorial Antibody that Rescues Leptin Deficiency In Vivo

Pingdong Tao, Yuanyuan Kuang, Yu Li, Wenping Li, Zibei Gao, Lili Liu, Min Qiang, Zhao Zha, Kun Fan, Peixiang Ma, Jeffrey M. Friedman, Guang Yang, and Richard A. Lerner**

Supplementary Figures and Tables:

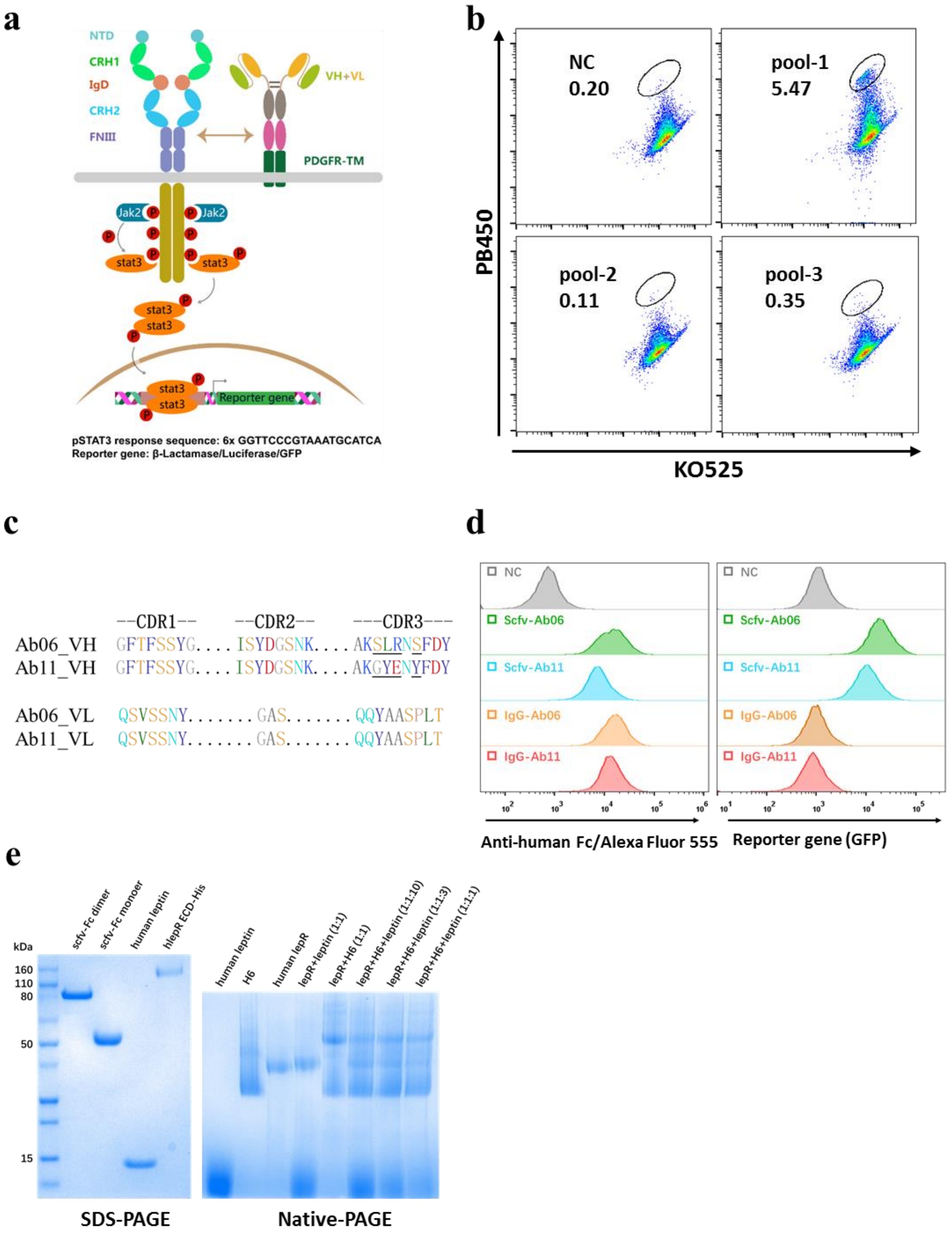

Figure S1. Characterization of agonist activity of combinatorial antibody ligands. a) Schematic illustration of a functional selection system for combinatorial antibody library based on STAT3 phosphorylation reporter gene assay; **b)** FACS sorting results of activated reporter cells infected with lentivirus containing a focused combinatorial antibody library; **c)** Sequence alignment of Ab06 and Ab11 in the CDR regions; **d)** Binding (left) and activation

(right) of scFv and full IgG to *h*LepR expressed reporter cell lines were analyzed by flow-cytometry; **e)** SDS- and native- PAGE analyses of H6, leptin, *h*LepR-ECD, and *h*LepR-ECD complexed with H6, leptin, or both at different ratios.

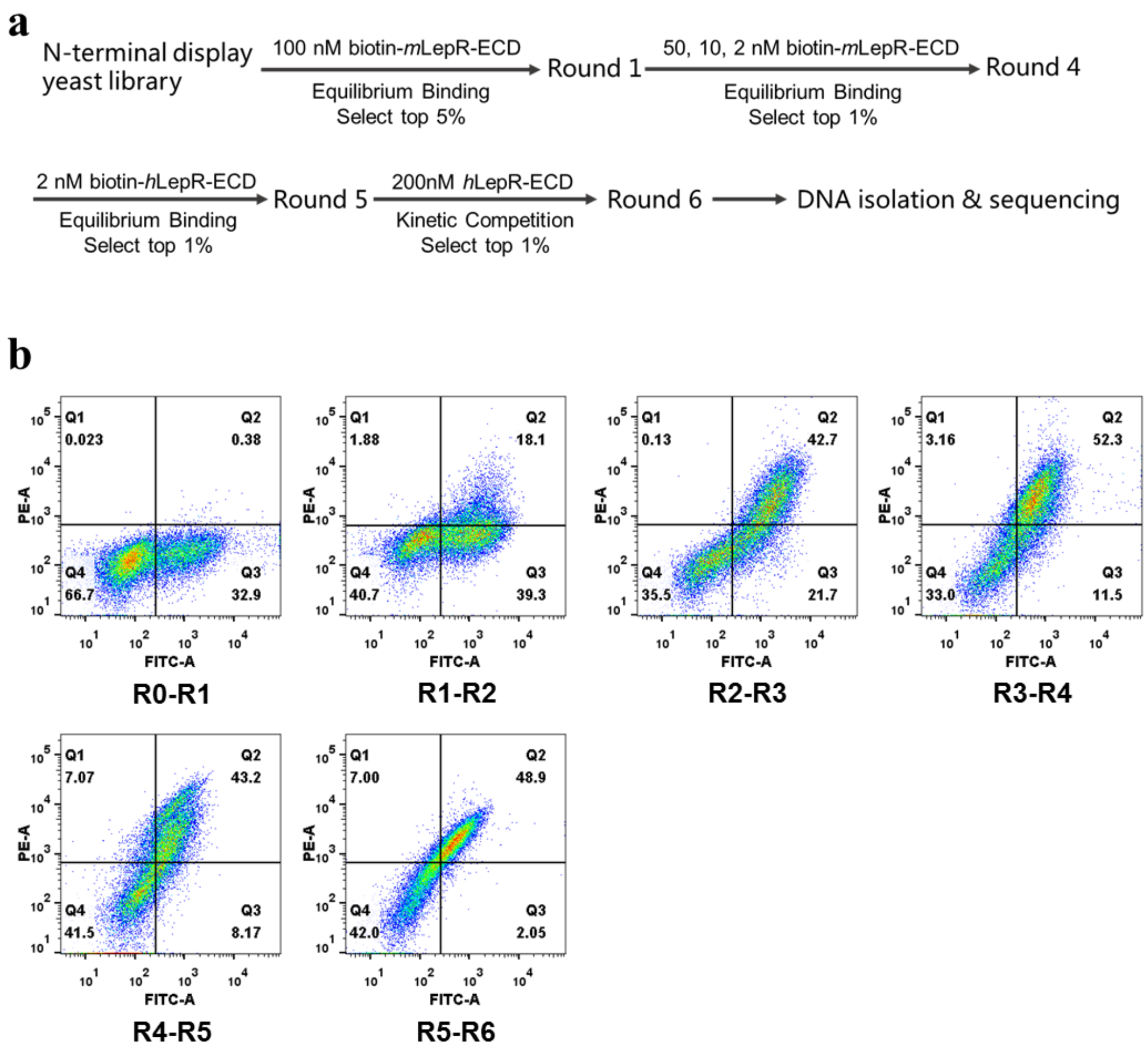

Figure S2. Affinity maturation of combinatorial antibodies using yeast surface display. a) Selection scheme of antibody affinity maturation using recombinant *h*LepR- and *m*LepR-ECD on yeast surface; **b)** Yeast sorting results of each selection round.

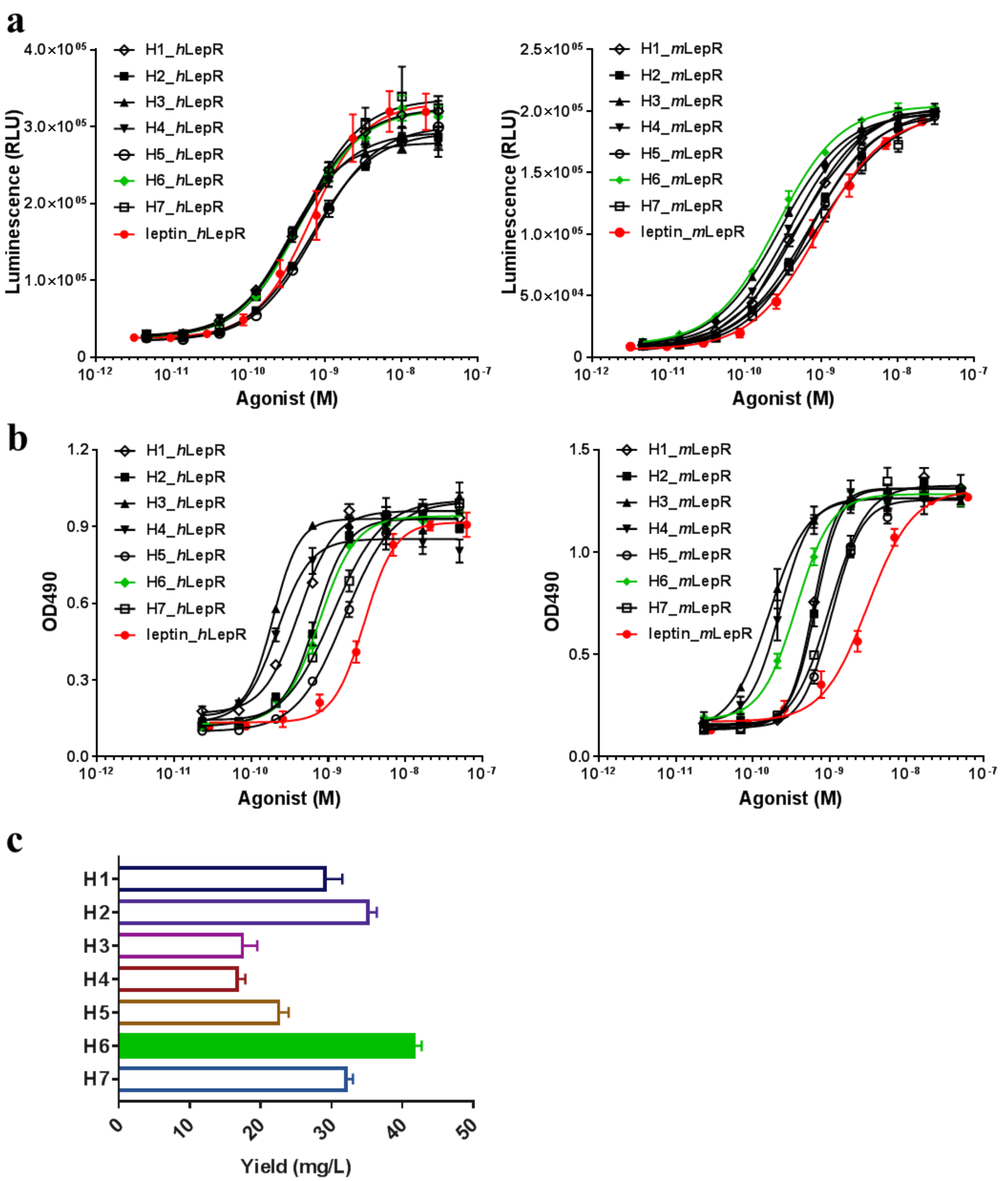

Figure S3. Affinity validation of combinatorial antibodies. a) Dose-dependent activation of LepR by leptin and high affinity agonist antibody candidates (H1-H7) *via* chemiluminescent detection of STAT3 phosphorylation in luciferase reporter cell lines (Luminescence); **b)** Dose-dependent activation of LepR by leptin and high affinity agonist antibody candidates (H1-H7) *via* leptin-dependent cell proliferation of murine IL-3 dependent Ba/F3 pre-B cells (OD490, absorption at wavelength 490 nm); **c)** Yields of scFv antibodies in 293F freestyle expression system. Data are shown as means ± SEM (error bars).

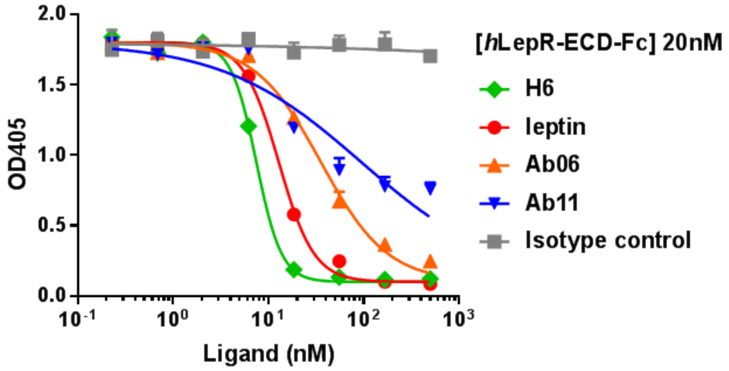

Ligand	IC_{50} (nM)
Leptin	13±0.43
H6	7.3±0.18
Ab06	40±1.4
Ab11	>100
Isotype control	N/A

Figure S4. Competitive ELISA assay for the binding of leptin and antibody against *h*lepR-ECD. Blocking of 20nM *h*LepR-Fc binding to coated leptin with different concentrations of leptin or antibody. Dose dependent competitive inhibition curve was fitted and IC50 of each ligand was calculated. All of the data are from three different experiments and shown as mean±SEM (error bars). N/A represents not applicable.

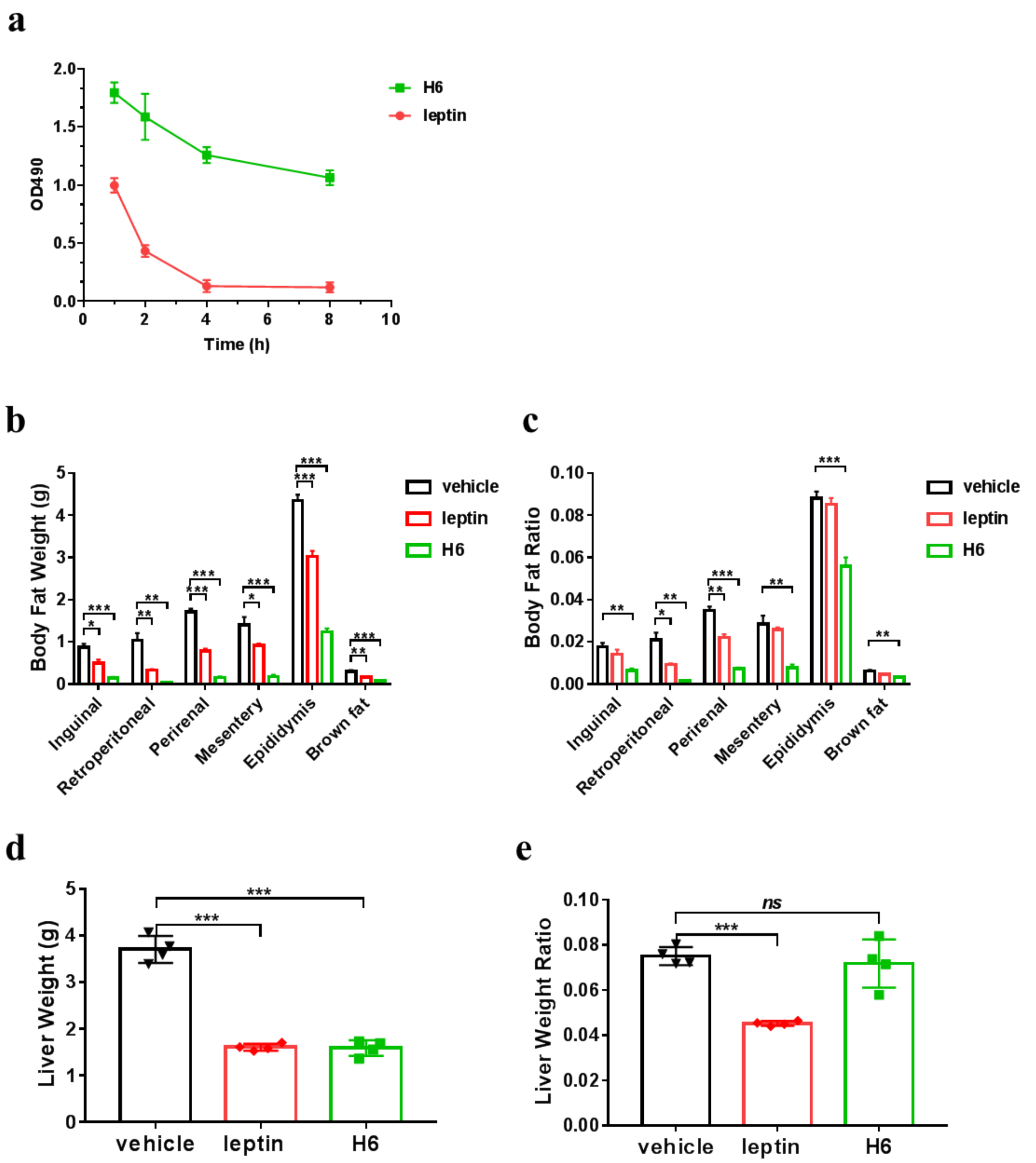

Figure S5. Pharmacokinetic studies and animal tissue samples analyses. a) A quick PK studies of leptin and antibody in wild type mice by intravenous injection. Leptin or antibody amounts were estimated at indicated time points based on Ba/F3 reporter cell proliferation assay; **b) & c)** Weight and weight ratio of adipose tissues in mice after leptin and H6 treatments, respectively; **d) & e)** Weight and weight ratio of livers in mice after leptin and H6 treatments, respectively. All of the data are shown as means ± SEM (error bars, n=8) by ANOVA, in which *ns* represents not significant. *P value <0.05, **P value < 0.01, ***P value < 0.001.

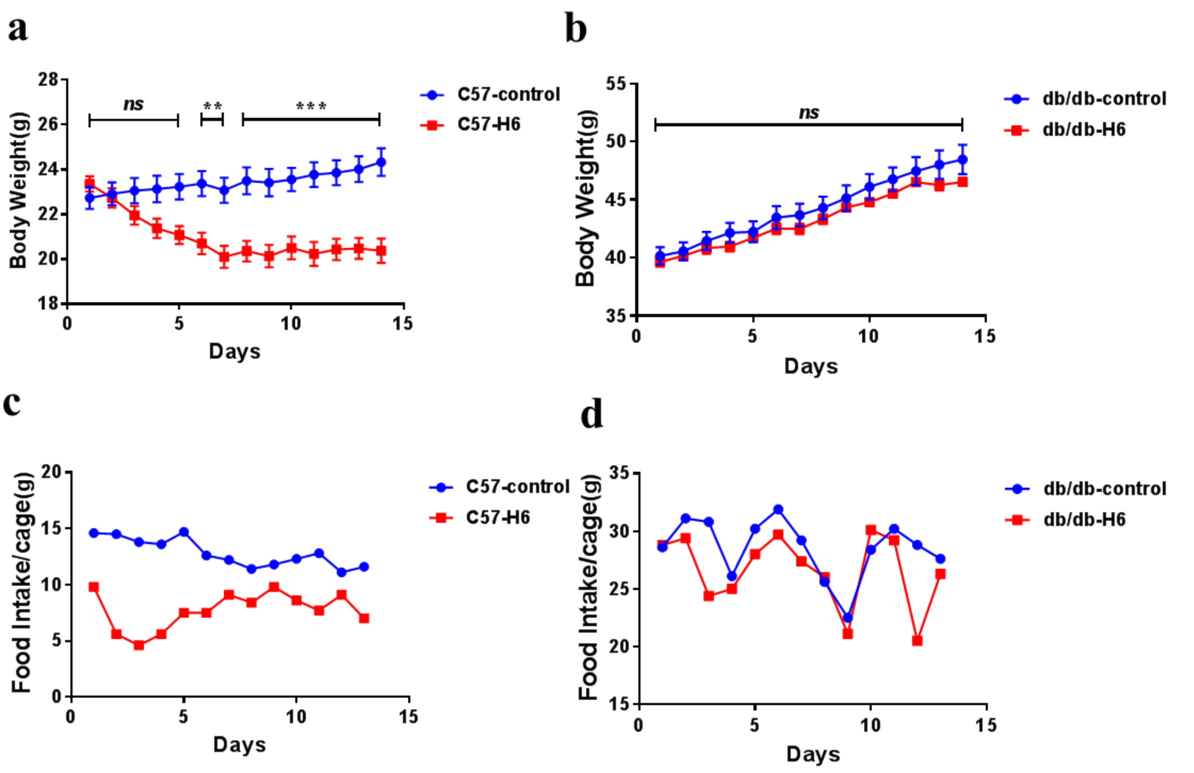

Figure S6. Antibody agonistic activity validation in wide type and *db*/*db* mice. a) & c) Daily body weight and food intake of treated wild type C57BL mice for two weeks; **b) & d)** Daily body weight and food intake of treated *db*/*db* mice. H6 group (n=5, 5.0 mg/kg, qod) and vehicle control group (n=5, 5.0 ml/kg, qod) contained 6 mice each. All of the data are shown as means ± SEM (error bars) by ANOVA, in which *ns* represents not significant. *P value <0.05, **P value < 0.01, ***P value < 0.001.

Table S1. Phage panning protocols for *h*LepR-focused combinatorial antibody libraries enrichment

Panning method	Antigen	Elution
POOL-1	*h*LepR-ECD-Fc dimer	glycine·HCl (pH2.0)
POOL-2	*h*LepR-ECD-Fc dimer	1 mg/mL leptin
POOL-3	*h*LepR-ECD-6xHis monomer	glycine·HCl (pH2.0)

Table S2. Amino acid sequences of five truncated motifs of *h*LepR-ECD

Motifs	Amino acid sequences
**NTD**	FNLSYPITPWRFKLSCMPPNSTYDYFLLPAGLSKNTSNSNGHYETAVEPKFNSS GTHFSNLSKTTFHCCFRSEQDRNCSLCADNIEGKTFVSTVNSLVFQ
**CRH1**	QIDANWNIQCWLKGDLKLFICYVESLFKNLFRNYNYKVHLLYVLPEVLEDSPL VPQKGSFQMVHCNCSVHECCECLVPVPTAKLNDTLLMCLKITSGGVIFQSPLM SVQPINMVKPDPPLGLHMEITDDGNLKISWSSPPLVPFPLQYQVKYSENSTTVI READKIVSATSLLVDSILPGSSYEVQVRGKRLDGPGIWSDWSTPRVFTTQDV
**IgD**	IYFPPKILTSVGSNVSFHCIYKKENKIVPSKEIVWWMNLAEKIPQSQYDVVSDH VSKVTFFNLNETKPRGKFTYDAVYCCNEHECHHRYAELYV
**CRH2**	IDVNINISCETDGYLTKMTCRWSTSTIQSLAESTLQLRYHRSSLYCSDIPSIHPISE PKDCYLQSDGFYECIFQPIFLLSGYTMWIRINHSLGSLDSPPTCVLPDSVVKPLP PSSVKAEITINIGLLKISWEKPVFPENNLQFQIRYGLSGKEVQWKMYEVYDAKS KSVSLPVPDLCAVYAVQVRCKRLDGLGYWSNWSNPAYTVVMD
**FNIII**	IKVPMRGPEFWRIINGDTMKKEKNVTLLWKPLMKNDSLCSVQRYVINHHTSC NGTWSEDVGNHTKFTFLWTEQAHTVTVLAINSIGASVANFNLTFSWPMSKVNI VQSLSAYPLNSSCVIVSWILSPSDYKLMYFIIEWKNLNEDGEIKWLRISSSVKK YYIHDHFIPIEKYQFSLYPIFMEGVGKPKIINSFTQDDIEKHQSD
